# Determination of Aflatoxin M1 and Chloramphenicol in Milk Based on Background Fluorescence Quenching Immunochromatographic Assay

**DOI:** 10.1155/2017/8649314

**Published:** 2017-03-06

**Authors:** Xiaoxia Wu, Xiaofeng Tian, Lihua Xu, Jiutong Li, Xinxia Li, Yuwen Wang

**Affiliations:** ^1^College of Pharmacy, Xinjiang Medical University, Urumqi 830000, China; ^2^Shanghai Simp Bio-Science Co., Ltd., Shanghai 201800, China; ^3^Center for Disease Control and Prevention, Xinjiang Uyghur Autonomous Region, Urumqi 830000, China

## Abstract

Harsh demanding has been exposed on the concentration of aflatoxin M1 (AFM1) and chloramphenicol (CAP) in milk. In this study, we developed a new method based on background fluorescence quenching immunochromatographic assay (bFQICA) to detect AFM1 and CAP in milk. The detection limit for AFM1 was 0.0009 ng/mL, while that for the CAP was 0.0008 ng/mL. The assay variability was determined with 3 AFM1 standards (i.e., 0.25 ng/mL, 0.5 ng/mL, and 1.0 ng/mL), and the actual detection value was 0.2497, 0.5329, and 1.0941, respectively. For the assay variability of 3 CAP standards (i.e., 0.10 ng/mL, 0.30 ng/mL, and 0.50 ng/mL), the actual detection value was 0.0996, 0.3096, and 0.4905, respectively. The recovery rate of AFM1 was 99.7%–101.7%, while that for CAP was 95.3%–97.6%. For the test stability, AFM1 and CAP showed satisfactory test stability even at month 5. Compared with the sensitivity of liquid chromatography-mass spectrometry (LC-MS) method, no statistical difference was noticed in results of the bFQICA. Our method is convenient for the detection of AFM1 and CAP in milk with a test duration of about 8 minutes. Additionally, an internal WiFi facility is provided in the system allowing for quick connection and storage in the intelligent cell phone.

## 1. Introduction

Feeding of drugs and chemicals to cattle can leave residues in milk and meat. For example, aflatoxin M1 (AFM1), the hydroxylated metabolite of aflatoxin B1, is a carcinogenic substance detected in milk and dairy products [1]. Application of chloramphenicol (CAP), a broad spectrum antibiotic frequently used in the husbandry for the prevention and management of certain diseases, may result in residue in the milk and dairy products [2]. Long-term intake of these products may induce drug-resistance and adverse reactions such as allergy [3]. On this basis, a harsh detection limit of less than 0.5 *μ*g/L has been issued by FDA and SFDA for AFM1 in the milk and dairy products, and CAP is listed as a forbidden chemical [4].

Currently, several methods have been developed for the detection of AFM1 and CAP, such as enzyme linked immunosorbent assay (ELISA), high performance liquid chromatography (HPLC), and gold immune chromatographic assay (GICA) [5–9]. These methods were reported to be applicable for the determination of AFM1 and CAP; however, their extensive application is hampered due to their limitations such as being time- and labor-intensive for ELISA, as well as limitations in the quantitative analysis of the sample using GICA. In the food safety testing, it is urgent to develop methods that are convenient and easy to perform, which allows for quick and instant determination.

Recently, a quantitative assay named background fluorescence quenching immune chromatographic assay has been developed based on the fluorescence quenching and nitrocellulose membrane background signals. The method has been used for the alpha fetoprotein in clinical practice. Unlike the conventional GICA assay, in the background fluorescence quenching immunochromatographic assay (bFQICA), the fluorescence donors are fluorescein that are precoated on the entire nitrocellulose membrane and quenching occurs between the gold particles and nitrocellulose membrane [10]. In this study, we firstly applied such technique in the food safety. More importantly, a modification was made on the preparation of the test strip which improved the reproducibility. Besides, an internal WiFi facility is provided in the system, which allows for quick connection and storage in the intelligent cell phone. On this basis, two test strips were developed for the detection of AFM1 and CAP in milk, respectively. The efficiency of the strips was assessed by evaluating the limit of blank and variability assay, test repeatability, and concentration recovery, as well as test stability.

## 2. Materials and Methods

### 2.1. Reagents

The bFQICA analyzer was provided by the Simp Bio-Science Co., Ltd. (Shanghai, China). The reference substance and the monoclonal antibody of AFM1 were purchased from Rohi Biotech Co., Ltd. (Shanghai, China). The AFM1-bovine serum albumin (BSA) conjugate, reference substance and the monoclonal antibody of CAP, CAP-BSA conjugate, and the goat anti-mouse IgG were purchased from Rohi Biotech Co., Ltd. (Shanghai, China).

### 2.2. Preparation of Gold Nanoparticles (GNPs)

GNPs were made through trisodium citrate reduction of hydrogen tetrachloroaurate (iii) hydrate (HAuCl4·3H_2_O) as previously described [11]. After preparation, the GNPs were subject cooled at room temperature, followed by sterilization and storage at 4°C.

### 2.3. Preparation of Test Strip

**Figure 1:**
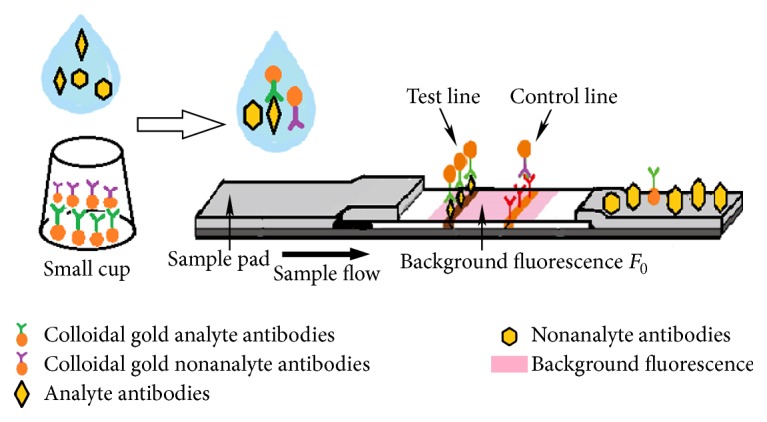
Diagram of test strip used in the background fluorescence quenching immunochromatographic assay (bFQICA).

The test strip consisted of sample pad, background fluorescence (*F*_0_), test line, and control line (Figure 1). Initially, the test samples were add to the small cup containing the GNPs-labeled antibody. On this basis, the AFM1 or CAP could specifically bind with the GNPs-labeled antibody. Afterwards, a portion of the mixture was dripped onto the sample pad. The samples could migrate in a direction towards the test line through the capillarity. Upon reaching the test line, the remanent GNPs-labeled antibody not binding with AFM1 or CAP bound with the coated antigen of AFM1 or CAP to form the antigen-antibody-GNP complex that quenches the fluorescence on the test line. The content of test samples was correlated to the quenching of the fluorescence presented as the ratio of fluorescence on the test line to the background fluorescence (*F*_1_/*F*_0_) [10].

For the preparation of GNP-labeled AFM1 antibody, AFM1 antibody was labeled by GNP solutions (pH 7.0) with a concentration of 0.30 *μ*g/mL, 0.60 *μ*g/mL, 0.90 *μ*g/mL, and 1.20 *μ*g/mL, respectively. For the preparation of GNP-labeled CAP antibody, CAP was labeled by GNP solution (pH 7.5) with a concentration of 0.30 *μ*g/mL, 0.60 *μ*g/mL, 0.90 *μ*g/mL, and 1.20 *μ*g/mL. Ten minutes later, the mixture (1 mL) was add to an Eppendorf tube, followed by adding 100 *μ*L PBS and incubating for 2 hrs. Afterwards, the mixture was centrifuged at 12,000 r/min for 10 min. The pellet was suspended in 3% bovine serum albumin (BSA) (1 mL) solution, and the supernatant was washed using PBS after centrifugation. Subsequently, the mixture was mixed with 0.5 *μ*g/mL gold based goat anti-mouse antibody (20 *μ*L). Finally, 10 *μ*L product was transferred into a test tube and dehydrated for 12 hrs under vacuum.

For the preparation of AFM1 or CAP coupled antibody, the AFM1-BSA was diluted using PBS buffer into a concentration of 0.30 mg/mL, 0.50 mg/mL, and 0.70 mg/mL, respectively, while that of CAP-BSA was 0.20 mg/mL, 0.40 mg/mL, and 0.60 mg/mL, respectively. Subsequently, the solution was coated onto the test line of the nitrocellulose membrane and kept at room temperature for 8 hrs. On this basis, three test strips coated with AFM1-BSA (0.30 mg/mL, 0.50 mg/mL, and 0.70 mg/mL) and CAP-BSA (0.20 mg/mL, 0.40 mg/mL, and 0.60 mg/mL) on the test line were obtained.

The reference substance of AFM1 (0.25 ng/mL, 0.50 ng/mL, 1.00 ng/mL, and 2.00 ng/mL) or CAP (0.10 ng/mL, 0.30 ng/mL, 0.50 ng/mL, and 1.00 ng/mL) diluted by PBS was add to each test tube. After that, 100 *μ*L each mixture was add to each test tube with gold probe based AFM1. Part of the mixture (60 *μ*L) was added onto the sample port on the test line to obtain the reading. The optimal density of GNP-labeled AFM1 antibody and concentration of AFM1 coupled antibody were obtained in presence of significant difference between* F*_1_/*F*_0_ at four concentrations.

### 2.4. Instrument Fabrication and Functions

**Figure 2:**
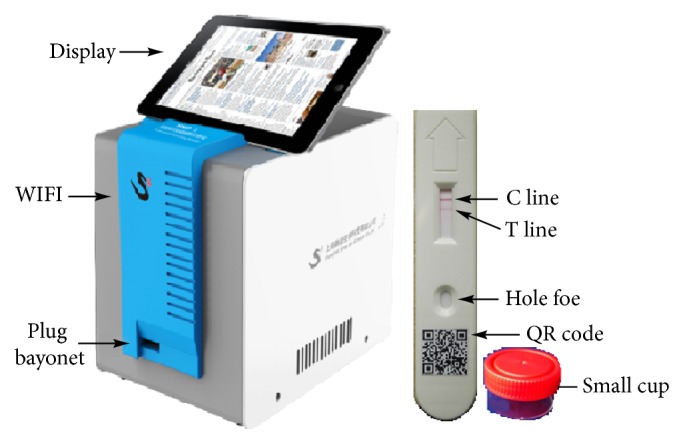
Fabrication of the bFQICA system.

The bFQICA reader consisted of several core parts including an optical sensor, a scanning platform, and the stepping system, as well as signal processing system (Figure 2). Briefly, 100 *μ*L test samples were add to the small cup containing GNPs-labeled antibody. Then 60 *μ*L mixture was dripped onto the hole foe (Figure 2) at room temperature for 8 min. Afterwards, the test strip was inserted into the plug bayonet, and the reading was* F*_1_/*F*_0_ of the test samples.

### 2.5. Establishing Standard Curve

Using serial dilutions of the standard solution, we established a standard curve for the analysis of known samples with AFM1 or CAP in the range of 0–2.0 ng/mL. In brief, 100 *μ*L of each mixture was add to each test tube with gold probe based AFM1 or CAP. Part of the mixture (60 *μ*L) was added onto the sample port on the test line to obtain the* F*_1_/*F*_0_. After reading, the curves were established using concentration of AFM1 or CAP standard as the *x*-axis and* F*_1_/*F*_0_ at each concentration as *y*-axis.

### 2.6. BFQICA Performance and Validations

Upon preparation of the test strips, AFM1 or CAP standards at various concentrations were used to assess the performance of bFQICA, including assay limit of blank and variability, test repeatability, and concentration recovery, as well as test stability. For the detection of AFM1 and CAP, 100 *μ*L milk was directly add to the small cup containing the GNP-labeled antibody, and then 60 *μ*L of mixture was added onto the test line.

### 2.7. LC-MS Assay

LC-MS detection was performed according to the conventional description. The samples were extracted using the methyl cyanide-Mcilvaine buffer and then subjected to the Eclipse XDB-C18 column (150 mm × 2.1 mm, 3.5 *μ*m). The flow speed was 0.25 mL/min.

## 3. Results

### 3.1. The Optimal Density of GNP-Labeled Antibody and Concentration of Coupled Antibody

**Figure 3:**
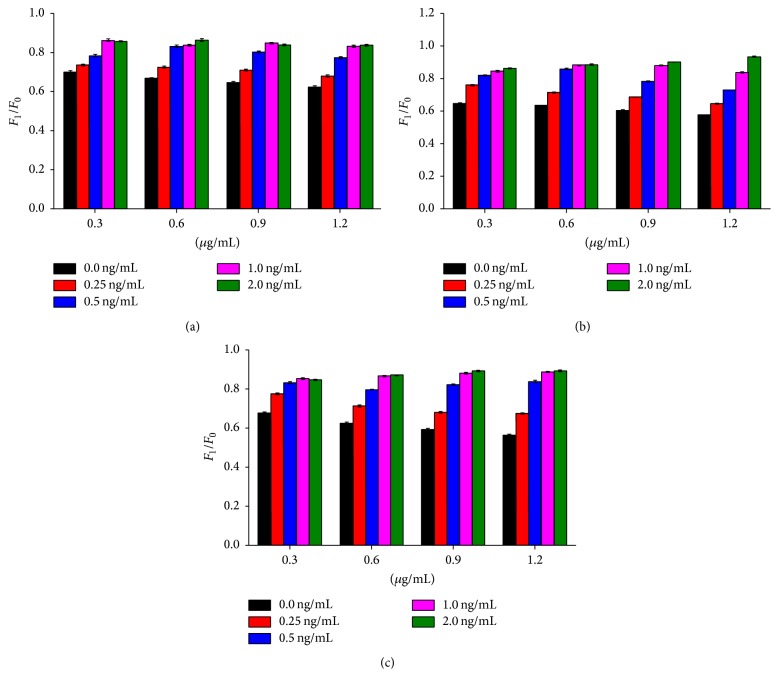
Determination of optimal density of GNP-labeled antibody and concentration of coupled antibody of AFM1. AFM1 antibody was labeled by GNP solutions (pH 7.0) with a concentration of 0.30 *μ*g/mL, 0.60 *μ*g/mL, 0.90 *μ*g/mL, and 1.20 *μ*g/mL. The antibody with a concentration of 0.3 mg/mL (a), 0.5 mg/mL (b), and 1.0 mg/mL (c) was used to select the optimal density of GNP-labeled AFM1 antibody and concentration of AFM1 coupled antibody, defined as presence of significant difference between* F*_1_/*F*_0_ at four concentrations.

**Figure 4:**
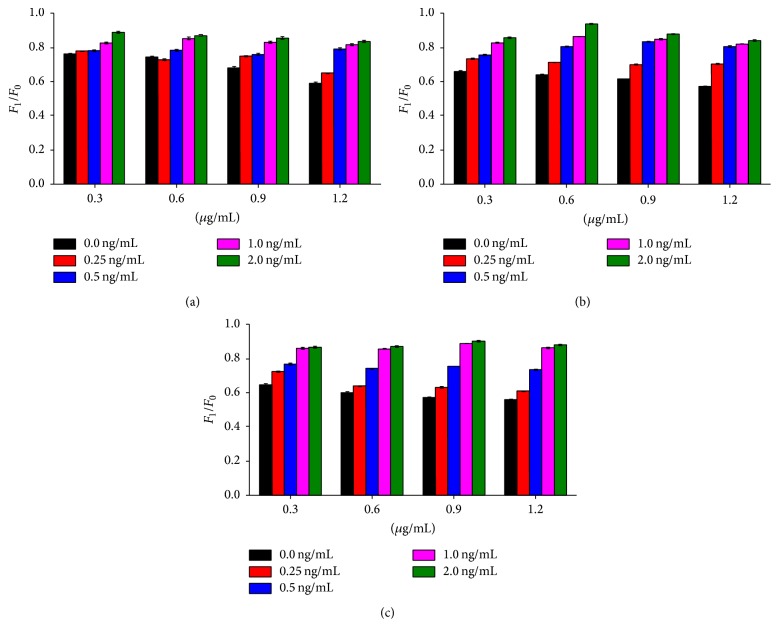
Determination of optimal density of GNP-labeled antibody and concentration of coupled antibody of CAP. CAP antibody was labeled by GNP solutions (pH 7.0) with a concentration of 0.30 *μ*g/mL, 0.60 *μ*g/mL, 0.90 *μ*g/mL, and 1.20 *μ*g/mL. The antibody with a concentration of 0.2 mg/mL (a), 0.4 mg/mL (b), and 0.6 mg/mL (c) was used to select the optimal density of GNP-labeled CAP antibody and concentration of coupled antibody, defined as presence of significant difference between* F*_1_/*F*_0_ at four concentrations.

In this study, the optimal density of GNP-labeled AFM1 antibody was 1.2 *μ*g/mL, and concentration of AFM1 coupled antibody was 0.5 *μ*g/mL. Remarkable difference was noticed in* F*_1_/*F*_0_ in presence of GNP-labeled AFM1 antibody of 1.2 *μ*g/mL and concentration of AFM1 coupled antibody of 0.5 *μ*g/mL (Figure 3). Remarkable difference was noticed in* F*_1_/*F*_0_ in presence of GNP-labeled CAP antibody of 0.6 *μ*g/mL and concentration of CAP coupled antibody of 0.4 *μ*g/mL. Thus, the optimal density of GNP-labeled CAP antibody in the small cup was 0.6 *μ*g/mL, and the concentration of CAP coupled antibody in the test line was 0.4 *μ*g/mL (Figure 4).

### 3.2. Establishing Standard Curves of AFM1 and CAP

The formula of AFM1 was as follows: *Y* = −1.7088*χ*^2^ + 1.5707*χ* + 0.5977 (*r* = 0.9966). For CAP, the formula was *Y* = −0.2519*χ*^2^ + 0.5353*χ* + 0.6558 (*r* = 0.9923), where *χ* is concentration of AFM1 or CAP standard solution and *Y* is the corresponding* F*_1_/*F*_0_.

### 3.3. Assay Limit of Blank and Variability

To test the limit of blank, a blank sample of AFM1 or CAP (0 ng/mL) was analyzed with the bFQICA system. The test was repeated 20 times, and a detection limit of 0.0009 ng/mL was obtained for AFM1, while that for the CAP was 0.0008 ng/mL. The assay variability was determined with 3 AFM1 standards at 0.25 ng/mL, 0.5 ng/mL, and 1.0 ng/mL, and the actual detection value was 0.2497 (RSD = 0.21%), 0.5329 (RSD = 0.13%), and 1.0941 (RSD = 0.15%), respectively. For the assay variability of 3 CAP standards at 0.10 ng/mL, 0.30 ng/mL, and 0.50 ng/mL, the actual detection value was 0.0996 (RSD = 1.76%), 0.3096 (RSD = 1.03%), and 0.4905 (RSD = 0.26%), respectively.

### 3.4. Test Repeatability

To assess the test repeatability of the system, the concentration of AFM1 and CAP in the milk obtained in a local supermarket was determined. The concentration of AFM1 was 0.008 ng/mL (RSD = 0.54%), while that for the CAP was 0.0011 ng/mL (RSD = 0.45%).

### 3.5. Concentration Recovery

The milk samples (1 mL) were add to an Eppendorf tube, followed by addition of 10 ng/mL AFM1 or CAP of a volume of 50 *μ*L, 30 *μ*L, and 10 *μ*L, respectively. Afterwards, the mixture was dripped on the sample port of the test strip. The recovery rate of AFM1 was 100.1% (RS = 0.20%), 99.7% (RS = 0.35%), and 101.7% (RS = 0.87%), respectively (Table 1). The recovery rate of CAP was 95.3% (RS = 0.60%), 97.7% (RS = 1.18%), and 97.6% (RS = 0.59%), respectively (Table 1).

### 3.6. Test Stability

In this section, we determined the test stability by AFM1 or CAP standard and the standard of the same batch at months 1, 2, 3, 4, and 5, respectively. Both AFM1 and CAP showed satisfactory test stability even at month 5 (Table 2).

### 3.7. Sensitivity Test

The test milk containing AFM1 or CAP was subject to bFQICA detection and LC-MS detection. The detected concentration of AFM1 and CAP was listed in Table 3. Compared with the LC-MS method, no statistical difference was noticed in the bFQICA (*P* > 0.05, Table 3).

## 4. Discussion

Food safety has been a great concern worldwide. Harsh demanding has been exposed on the concentration of AFM1 and CAP in milk. To date, several methods have been developed for the detection of these drugs such as ELISA and LC-MS. In this study, we developed a new method for the detection of AFM1 and CAP in milk which was more convenient with high specificity and sensitivity.

The presence of AFM1 and CAP in milk has been a concern in some countries, which promotes the emergence of determination of these chemicals using various methods, such as ELISA, HPLC, and immunochromatographic assay. For example, in a previous study [12], Behfar et al. determined 100 samples of pasteurized milk from a local factory, which revealed that the concentration of AFM1 was ranged from 0.45 to 9.760 ng/L, which was below the accepted level (50 ng/L) in milk in Iran. Meanwhile, in a study in which AFM1 levels in samples were analyzed with a commercial competitive ELISA kit and HPLC, the quantification limit was 10 ng/L for ELISA combined with HPLC [13]. For the determination of CAP residues in milk, Wang et al. reported that the detection was 0.042 ± 0.006 ng/mL using an ELISA-based method designated as sensitive biotin-streptavidin amplified ELISA method [14]. For the immunochromatographic assay involving GNPs, Byzova et al. revealed that the detection limit of CAP in the milk was 10 ng/mL [15]. Compared with the previous studies which were with satisfactory efficiency or detection limit but were labor- or time-intensive, the study [15] reported that the assay duration was 10 min and could be carried out at room temperature without any additional devices and reactants. Also, the developed test strips have been used in the detection of CAP in dairy products.

Using this method, we confirmed that the AFM1 antigen coating concentration was 0.5 mg/mL in the test line, and the corresponding GNP-labeled AFM1 antibody concentration in the test tube was 1.2 *μ*g/mL, while that for the CAP was 0.4 mg/mL and 0.6 *μ*g/mL, respectively. Meanwhile, method validation was also confirmed in our study. The test efficiency showed no difference compared with the standard method proposed by SFDA. The detection method was stored in the two-dimensional codes, and quantitative test strips were established for CAP and AFM1, respectively. For the sensitivity, compared with the LC-MS method that had been considered as the golden standard with high accuracy, no statistical difference was noticed in the results obtained from bFQICA. This validated the accuracy of our method.

As is known to all, false positive samples were noticed which may be related to the application of such technique and enzyme labeling [16, 17]. The facilities used in the HPLC and UPLC-MS were highly expensive, which hampered their extensive application [18, 19]. On this basis, it is necessary to develop new methods with features of quick detection with higher sensitivity and specificity. In this study, we developed a method for the detection of CAP and AFM1 in milk and developed test strips accordingly. The bFQICA used in this study involved the immunochromatographic assay based on the combination of antibody and antigen, which shows high specificity. Using this method, the qualitative analysis manifested as the color changes induced by GNP accumulation and quantitative analysis manifested as fluorescence ratio between nitrocellulose membrane backgrounds and specific signals could be achieved simultaneously. Compared with the GICA method which may present inadequate reaction after binding of tested solution and the GNP antibody, the bFQICA method is more specific and precise as the test solution and the GNP antibody is subject to a complete reaction in the test tube, followed by dripping on the test strip. The detection limit is considered as an important parameter for a certain method when detecting a substance. For instance, Picinin et al. reported that the detection limit of AFM1 in milk was up to 0.15 ng/mL using the UPLC-MS method [20]. Nicolich et al. revealed that the detection limit of CAP in milk was 0.05 ng/mL using ELISA [21]. Using the test strips, the detection limit of AFM1 and CAP was 0.0009 ng/mL and 0.0008 ng/mL, respectively. Besides, the test duration was about 8 minutes. Also, we determined the test efficiency of our method compared with the LC-MS, which showed no statistical difference.

## 5. Conclusions

In this study, we developed a new method for the detection of AFM1 and CAP in milk which was more convenient with high specificity and sensitivity. The detection limit for AFM1 was 0.0009 ng/mL, while that for the CAP was 0.0008 ng/mL. Besides, the method showed satisfactory stability and test efficiency.

## Figures and Tables

**Table 1:** Recovery rate of AFM1 and CAP.

Sample	Actual value (ng)	Measured value (ng)	Recovery rate (%)	RSD (%)
AFM1	0.25	0.2503	100.1	0.20
	0.50	0.4994	99.7	0.35
	1.00	1.0167	101.7	0.87

CAP	0.10	0.0953	95.3	0.60
	0.30	0.2933	97.7	1.18
	0.50	0.4883	97.6	0.59

AFM1, aflatoxin M1; CAP, chloramphenicol; RSD, relative standard deviation.

**Table 2:** Test stability of the strip for AFM1 and CAP.

Concentration		*F*_1_/*F*_0_ at month 1	*F*_1_/*F*_0_ at month 2	*F*_1_/*F*_0_ at month 3	*F*_1_/*F*_0_ at month 4	*F*_1_/*F*_0_ at month 5	RSD (%)
AFM1	0.00	0.5996	0.5892	0.5930	0.6004	0.5915	0.84
	0.25	0.6594	0.6588	0.6479	0.6581	0.6482	0.89
	0.50	0.7184	0.7091	0.7122	0.7093	0.7159	0.57
	1.00	0.8458	0.8513	0.8475	0.8434	0.8522	0.43
	2.00	0.9416	0.9425	0.9368	0.9570	0.9485	0.82

CAP	0.0	0.6531	0.6508	0.6549	0.6528	0.6545	0.25
	0.1	0.7063	0.7122	0.7051	0.7066	0.7081	0.39
	0.3	0.8054	0.8031	0.8024	0.8105	0.8062	0.40
	0.5	0.8509	0.8473	0.8516	0.8526	0.8479	0.27
	1.0	0.9406	0.9380	0.9415	0.9402	0.9411	0.14

AFM1, aflatoxin M1; CAP, chloramphenicol; RSD, relative standard deviation.

**Table 3:** Comparison of test efficiency between LC-MS and bFQICA.

Sample	bFQICA (*μ*g/kg)	LC-MS (*μ*g/kg)
AFM1 in sample 01	0.1151	0.0988
AFM1 in sample 02	0.1492	0.151
CAP in sample 03	0.1768	0.160
CAP in sample 04	0.4925	0.500

bFQICA, background fluorescence quenching immunochromatographic assay; LC-MS, liquid chromatography-mass spectrometry. Two samples were used for the AFM1 (samples 01 and 02) and CAP (samples 03 and 04), respectively. No statistical difference was noticed between the efficiency of bFQICA and LC-MS.

## References

[1] Prandini A., Tansini G., Sigolo S., Filippi L., Laporta M., Piva G. (2009). On the occurrence of aflatoxin M1 in milk and dairy products. *Food and Chemical Toxicology*.

[2] Su P., Liu N., Zhu M. (2011). Simultaneous detection of five antibiotics in milk by high-throughput suspension array technology. *Talanta*.

[3] Liu N., Su P., Gao Z. (2009). Simultaneous detection for three kinds of veterinary drugs: chloramphenicol, clenbuterol and 17-beta-estradiol by high-throughput suspension array technology. *Analytica Chimica Acta*.

[4] Jiang W., Wang Z., Nölke G., Zhang J., Niu L., Shen J. (2013). Simultaneous determination of aflatoxin B1 and aflatoxin M1 in food matrices by enzyme-linked immunosorbent assay. *Food Analytical Methods*.

[5] Tao X., Jiang H., Zhu J. (2014). An ultrasensitive chemiluminescent ELISA for determination of chloramphenicol in milk, milk powder, honey, eggs and chicken muscle. *Food and Agricultural Immunology*.

[6] Li X., Li P., Zhang Q. (2013). A sensitive immunoaffinity column-linked indirect competitive ELISA for ochratoxin a in cereal and oil products based on a new monoclonal antibody. *Food Analytical Methods*.

[7] Razzazi-Fazeli E., Noviandi C. T., Porasuphatana S., Agus A., Böhm J. (2004). A survey of aflatoxin B1 and total aflatoxin contamination in baby food, peanut and corn products sold at retail in Indonesia analysed by ELISA and HPLC. *Mycotoxin Research*.

[8] Urusov A. E., Zherdev A. V., Dzantiev B. B. (2014). Use of gold nanoparticle-labeled secondary antibodies to improve the sensitivity of an immunochromatographic assay for aflatoxin B1. *Microchimica Acta*.

[9] Wu C.-C., Lin H.-Y., Wang C.-P. (2015). Evaluation of a rapid quantitative determination method of PSA concentration with gold immunochromatographic strips Urological Oncology. *BMC Urology*.

[10] Chen X., Xu Y., Yu J. (2014). Antigen detection based on background fluorescence quenching immunochromatographic assay. *Analytica Chimica Acta*.

[11] Frens G. (1973). Controlled nucleation for the regulation of the particle size in monodisperse gold suspensions. *Nature Physical Science*.

[12] Behfar A., Khorasgani Z. N., Alemzadeh Z., Goudarzi M., Ebrahimi R., Tarhani N. (2012). Determination of aflatoxin M1 levels in produced pasteurized milk in ahvaz city by using HPLC. *Jundishapur Journal of Natural Pharmaceutical Products*.

[13] Radoi A., Targa M., Prieto-Simon B., Marty J.-L. (2008). Enzyme-linked immunosorbent assay (ELISA) based on superparamagnetic nanoparticles for aflatoxin M1 detection. *Talanta*.

[14] Wang L., Zhang Y., Gao X., Duan Z., Wang S. (2010). Determination of chloramphenicol residues in milk by enzyme-linked immunosorbent assay: improvement by biotin-streptavidin-amplified system. *Journal of Agricultural and Food Chemistry*.

[15] Byzova N. A., Zvereva E. A., Zherdev A. V., Eremin S. A., Dzantiev B. B. (2010). Rapid pretreatment-free immunochromatographic assay of chloramphenicol in milk. *Talanta*.

[16] Schneider R. S., Lindquist P., Tong-in Wong E., Rubenstein K. E., Ullman E. F. (1973). Homogeneous enzyme immunoassay for opiates in urine. *Clinical Chemistry*.

[17] Keller A., Eng J., Zhang N., Li X.-J., Aebersold R. (2005). A uniform proteomics MS/MS analysis platform utilizing open XML file formats. *Molecular Systems Biology*.

[18] Shackleton C. (2010). Clinical steroid mass spectrometry: a 45-year history culminating in HPLC–MS/MS becoming an essential tool for patient diagnosis. *Journal of Steroid Biochemistry and Molecular Biology*.

[19] Mensch J., Noppe M., Adriaensen J. (2007). Novel generic UPLC/MS/MS method for high throughput analysis applied to permeability assessment in early Drug Discovery. *Journal of Chromatography B: Analytical Technologies in the Biomedical and Life Sciences*.

[20] Picinin L. C. A., Cerqueira M. M. O. P., Vargas E. A., Lana Â. M. Q., Toaldo I. M., Bordignon-Luiz M. T. (2013). Influence of climate conditions on aflatoxin M1 contamination in raw milk from Minas Gerais State, Brazil. *Food Control*.

[21] Nicolich R. S., Werneck-Barroso E., Marques M. A. S. (2006). Food safety evaluation: detection and confirmation of chloramphenicol in milk by high performance liquid chromatography-tandem mass spectrometry. *Analytica Chimica Acta*.

